# Assessment of Knowledge, Attitude, and Practice of First Aid Management of Choking Among Primary School Teachers in Riyadh, Saudi Arabia: A Cross-Sectional Study

**DOI:** 10.7759/cureus.51519

**Published:** 2024-01-02

**Authors:** Mazyad M Alenezi, Zinab H Bohulaigah, Nader F Aldajani, Lamya G Alotaibi, Mthayel F Alshammari

**Affiliations:** 1 Otolaryngology - Head and Neck Surgery, Qassim University, Qassim, SAU; 2 College of Medicine, Almaarefa University, Riyadh, SAU; 3 Otolaryngology - Head and Neck Surgery, King Fahad Medical City, Riyadh, SAU

**Keywords:** choking, airway foreign body, heimlich maneuver, primary school teachers, pediatric first aid

## Abstract

Background: Understanding first aid is crucial for immediate intervention during health emergencies, with choking representing a significant danger, particularly for young children. Obstructed airways commonly lead to choking incidents, carrying substantial risks if not swiftly dealt with. This research endeavors to evaluate the comprehension, perspectives, and implementation of first aid measures for choking incidents among primary school teachers in Riyadh, Saudi Arabia, an area of paramount importance with minimal existing research within this particular demographic.

Methodology: This was a cross-sectional study conducted from the beginning of July till the end of October 2023, among 447 primary school teachers in Riyadh, Saudi Arabia. Data collection was carried out by administering a questionnaire through an online platform. The questionnaire included demographic information, knowledge about signs and symptoms of choking, the attitude of participants, participant’s practice, and the relationship between the knowledge level about choking and practice. The data collected were reviewed, coded, and then fed into IBM SPSS Statistics for Windows, Version 29 (Released 2023; IBM Corp., Armonk, New York, United States).

Results: Our study on first aid management of choking among primary school teachers highlighted significant findings. Participants demonstrated high awareness of choking signs with 386 (86.3%) recognizing universal signs and 330 (73.8%) claiming proficiency in first aid. Attitudes favored the importance of immediate treatment in 394 participants (88.1%) and the necessity of first aid knowledge for teachers (92.2%). One-hundred and fifty-one participants (33.8%) reported performing choking first aid with 328 (73.4%) opting for the Heimlich maneuver for a six-year-old. Significantly, higher knowledge correlated with increased first aid performance (p < 0.001) and support for mandatory training (p < 0.001).

Conclusion: Our study indicates a higher knowledge level in primary school teachers with positive attitudes and practices regarding choking first aid management. It underscores the importance of enhancing first aid training among primary school teachers, emphasizing its positive impact on choking management and the necessity of immediate intervention in such cases.

## Introduction

First aid is defined as an assessment and intervention that can be performed immediately by a person nearby with little or no medical equipment [1]. In circumstances where accidents occur in schools, providing urgent prehospital treatment is critical, with instructors frequently serving as first responders [2]. The purpose of first aid is to prevent or reverse potential harm as soon as possible before reaching the right healthcare facility. First aid knowledge consists of the procedures and skills needed to carry out actions linked to health emergency response and prevention [1]. In addition to assessing first aid knowledge, it is critical to analyze attitudes and conduct toward first aid provision [3].

Children are more vulnerable to injury and have a higher risk due to developmental and behavioral characteristics such as ignorance of hazards and being active, as well as physical characteristics such as smaller body mass and thinner, more vulnerable skin [4]. For children older than one year, choking can be treated with a mix of abdominal push and back blows using basic first aid techniques. The Heimlich maneuver, often called as abdominal thrust, is a highly successful procedure for clearing a foreign object-obsessed airway. The term "choking" refers to the inability to breathe when food or other foreign objects obstruct the upper airways. It is a true life-threatening emergency, and everyone in the victim's vicinity needs to act quickly to save their lives. Breathing difficulties may arise from an acute obstruction of the upper airway by foreign substances [2]. One of the main causes of injury-related morbidity and death is choking [5]. Toys, coins, and food are the main causes of choking-related incidents involving children that result in injury or death. Certain toys and foods have a higher risk of causing choking incidents among children due to their specific features, such as size, shape, and consistency. Comprehensive and well-coordinated preventive measures should be used to address the risk of choking hazards for children [6].

Most first aid is administered by ordinary individuals, with many people equipped to provide basic medical care and others ready to do so based on knowledge they have obtained. Furthermore, there are several scenarios that may necessitate medical assistance, and numerous nations have legislation, guidance, or direction that establishes a basic level of emergency treatment setup in specified conditions. This may include specific planning or having certain equipment available in the workplace [7]. It is imperative to remove the foreign body right away to avoid hypoxia-induced cardiac arrest. Better neurological outcomes following the removal were reported in a prior observational study [8]. Regretfully, it might take a while for an ambulance to arrive. Thus, in order to save lives, first aid programs must be made available to the general public in all nations, regardless of finances [9]. ‏It is estimated that 10-25% of injuries to children occur while they are in school [10]. Essential information and understanding to begin with help can be important for both instructors and understudies to be able to supply crisis care within the occasion of a mischance, conceivably sparing lives and minimizing damage in school settings [11]. Every teacher should be equipped with first aid techniques so that he/she can handle basic emergencies in the classroom. Every school should have standard operating procedures based on the school's requirements and teachers should be trained well in first aid response [12]. Additionally, in order to carry out the proper intervention, first aid providers need to be able to recognize and evaluate the severity of the illness or injury. As a result, it is crucial to teach schoolteachers the fundamentals of first aid administration and knowledge [13,14].

As a matter of fact, foreign body ingestion and aspiration cause a considerable amount of morbidity and mortality because of choking; it is the fourth most common cause of unintentional death in children under three and the third youngest among those under one year old [15].

Since there are only a few studies that have focused on a specific population such as primary school teachers, our goal for this study is to find out the knowledge, attitude, and practice of first aid management of choking among primary school teachers in Riyadh region, Kingdom of Saudi Arabia.

## Materials and methods

Study design and participants

We utilized a cross-sectional study design. The study was conducted from the beginning of July till the end of October 2023 to assess the knowledge, attitude, and practice of first aid management of choking among primary school teachers.

Inclusion and exclusion criteria

The study included primary school teachers who were willing to participate and had completed the entire questionnaire. individuals who declined to participate and those who did not complete the full questionnaire were excluded from the study. 

Sample size

All participants who met the inclusion criteria and fully completed the questionnaire were considered study participants. Thus, the sample size of the study was calculated using the Raosoft sample size calculator (Raosoft Inc, Seattle, WA) with a 95% confidence interval and a margin of error of 5%. The study used a sample size of 447 participants.

Sampling technique

We adopted a simple random sampling technique to ensure every individual in the target population had an equal and independent chance of being selected for the study. We adopted this technique to eliminate bias and allow better generalization of research findings to the entire population from which the sample is drawn.

Data collection

The data collection tool (questionnaire) was developed using Google Forms via social media platforms. The questionnaire consists of five domains. The first domain is socioeconomic, which includes questions regarding gender, age, educational level, nationality, marital status, and year of experience. The second domain is the assessment of knowledge of signs/symptoms of choking and its management. The third domain is the attitude of participants toward first aid management of choking. The fourth domain is about participants’ practice in first aid management of choking. The fifth domain is the relationship between the knowledge level about choking and the practice of choking first aid. The questionnaire was tested and exhibited satisfactory construct validity. The validation of the questionnaire in relation to the referenced article [2] confirms its validity, strong reliability, acceptable internal consistency, and excellent content validity. An extensive review of the tools used and approved by other researchers and consultation with the experts to include only questions that address the problem under study. The survey was distributed via various social media platforms, including X (formerly known as Twitter), Telegram, and WhatsApp. Each participant was asked to click on the consent statement before proceeding to complete the questionnaire. 

Data analysis

The data collected were reviewed, coded, and then fed into Windows, Version 29 (Released 2023; IBM Corp., Armonk, New York, United States) in frequencies and percentages, and quantitative data were presented in mean and standard deviation (SD). A suitable statistical test was used. A chi-square test was performed to test for differences in the proportions of categorical variables between two or more groups. p<0.05 was considered the cutoff value for statistical significance. All statistical tests used were two-tailed with an alpha level of 0.05. This was considered significant if the p-value was less than or equal to 0.05.

Ethical considerations 

Ethical approval was obtained from the ethics committee at Qassim University, Qassim, Saudi Arabia (approval number: IRB-25-57-03). Information was provided to all participants prior to obtaining consent. Before collecting data, informed consent was obtained from the participants. Furthermore, confidentiality was ensured for all interviewed participants. The data of each participant were taken in a re-identifiable manner. The data collected were stored electronically in electronic files, and no access was granted to anyone who was not a legitimate participant in the study.

## Results

Our study included 447 participants assessed for first aid choking management. Most participants were female 338 (75.6%), aged 40-50 years 170 (38%), married 346 (77.4%), and Saudi 414 (92.6%), with varying educational backgrounds including diploma 97 (21.7%), bachelor's 268 (60.0%), and master's degrees 51 (11.4%). A significant portion had over 10 years of teaching experience 263 (58.8%) (Table 1).

**Table 1:** Sociodemographic of all participants assessed for knowledge, attitude, and practice of first aid choking management N represents the frequency count of participants, while % represents the percentage that frequency accounts for out of the total data.

Sociodemographic		N=447	%
Gender	Female	338	75.6
	Male	109	24.4
Age	20-30 Years	85	19.0
	30-40 Years	139	31.1
	40-50 Years	170	38.0
	> 50 Years	53	11.9
Nationality	Non-Saudi	33	7.4
	Saudi	414	92.6
Education	Diploma	97	21.7
	Bachelor's Degree	268	60.0
	Master's Degree	51	11.4
	Others	31	6.9
Marital Status	Single	83	18.6
	Married	346	77.4
	Divorced/Widow	18	4.0
Years of Experience	< 1 Years	74	16.6
	1-5 Years	50	11.2
	5-10 Years	60	13.4
	> 10 Years	263	58.8

Table 2 assesses the participant's knowledge which demonstrated significant awareness of universal signs of choking inability to speak/breathe/swallow 386(86.3%), common behaviors leading to choking e.g., talking/laughing while eating 365 (81.6%), signs of complete airway obstruction e.g., can't cry/talk/breathe/cough 338 (75.6%), and signs of partial airway obstruction e.g., unable to talk 307 (68.6%), while 330 (73.8%) claimed proficiency in providing first aid, with learning sources primarily including social media 210 (47%) and previous first aid training 81 (18.1%). Previous first aid training was reported by 155 (34.7%), while only 174 (38.9%) expressed confidence. 

**Table 2:** Assessment of knowledge of signs/symptoms of choking and its management N represents the frequency count of participants, while % represents the percentage that frequency accounts for out of the total data.

Knowledge of Signs/Symptoms of Choking and Its Management		N=447	%
Universal Signs of Choking	Coughing/Crying	61	13.6
	Knocking at Chest	148	33.1
	Cinching at Throat	171	38.2
	Wheezing	99	22.1
	Inability to Speak/Breathe/Sallow	386	86.3
Behavior Leads to Choking	Playing while eating	301	67.3
	Talking/Laughing while eating	365	81.6
	Putting Objects in Their Mouth	330	73.8
	Playing Football	26	5.8
	Not Chewing Well	231	51.6
Signs/Symptoms of Complete Airway Obstruction	Crying Loud	21	4.6
	Coughing	70	15.6
	Can’t cry/talk/breathe/cough	338	75.6
	Blood in Sliva	39	8.7
	Wheezing	159	35.5
Signs/Symptoms of Partial Airway Obstruction	Unable to Talk	307	68.6
	Coughing	51	11.4
	Unable to Breathe	256	57.2
	Sneezing	14	3.1
	Unable to Swallow	179	40.0
Do you know how to Provide First Aid to Choking	No	117	26.2
	Yes	330	73.8
Where You Learned From	From Previous First Aid Training	81	18.1
	From a Friend	24	5.4
	From social media	210	47.0
	From Professionals	59	13.2
	Others	73	16.3
Previously Received First Aid Training for Choking	No	292	65.3
	Yes	155	34.7
How Confident You Are to Give First Aid Training	Confident	174	38.9
	Don't Know	176	39.4
	Not Sure	97	21.7

Table 3 shows the attitudes of participants toward first aid management of choking. A significant proportion strongly agreed that asphyxia must be treated immediately (88.1%) and that teachers should have knowledge of first aid for choking 412 (92.2%). Similarly, a high agreement was noted for the belief that not performing first aid is life-threatening 398(89%) and that first aid for choking can be administered at school 263 (81.2%). Moreover, participants largely agreed with the notion that it is not possible to begin first aid for choking without prior knowledge 196 (43.8%). However, opinions were divided on whether to call the ambulance team before performing first aid 177 (39.6%) strongly agreed.

**Table 3:** Attitude of participants toward first aid management of choking N represents the frequency count of participants, while % represents the percentage that frequency accounts for out of the total data.

		Strongly Disagree	Disagree	Not Sure	Agree	Strongly Agree
Asphyxia Must Be Treated Immediately	N	1	3	4	45	394
	%	0.2	0.7	0.9	10.1	88.1
Teachers Should have Knowledge of First Aid for Choking	N	1	1	1	32	412
	%	0.2	0.2	0.2	7.2	92.2
Not Performing First Aid is Life Threatening	N	1	1	4	43	398
	%	0.2	0.2	0.9	9.6	89.0
First Aid for Choking Can Be Done at School	N	-	1	10	73	363
	%	-	0.2	2.2	16.3	81.2
Not possible to Begin First Aid for Choking Without Prior Knowledge	N	6	56	44	145	196
	%	1.3	12.5	9.8	32.4	43.8
Call the Ambulance Team Before Performing First Aid for Choking	N	23	101	36	110	177
	%	5.1	22.6	8.1	24.6	39.6

Table 4 shows participants' practices concerning first aid management of choking. One-hundred and fifty-one (33.8%) reported performing choking first aid, while 368 (82.3%) advocated for teachers to attend a first aid course. In the event of a choking incident involving a six-year-old unable to cry, speak, or cough, 328 (73.4%) would perform the Heimlich maneuver. For an eight-year-old able to speak but with an ineffective cough, 216 (48.3%) would perform the Heimlich maneuver. In the case of an unresponsive, nine-year-old child, 195 (43.6%) would call an ambulance and start CPR.

**Table 4:** Participant’s practice about first aid management of choking N represents the frequency count of participants , while % represents the percentage that frequency accounts for out of the total data.

Participant’s Practice		N=447	%
Ever Performed Choking First Aid Inside/Outside of School	Yes	151	33.8
	Don't Know	26	5.8
	No	270	60.4
Support to Make It Necessary for Teachers to Attend Course of First Aid for Choking	Strongly Disagree	3	0.7
	Disagree	4	0.9
	Not Sure	6	1.3
	Agree	66	14.8
	Strongly Agree	368	82.3
Choking Incident of Six-Year-Old, Unable to Cry, Talk, Breathe, or Cough, Next Step You Perform	Ask child to cough& try out the foreign object	67	15.0
	Call emergency services and wait for help to arrive	26	5.8
	Encourage the child to breathe deeply to try to clear his airways	15	3.4
	Offer the child water or another drink to help him remove the foreign body	11	2.5
	Perform abdominal compressions (Heimlich maneuver) on the child to help clear the airway	328	73.4
Choking Incident, Eight-Year-Old, Able to Speak, Cough Ineffective, Next Step You Perform	Ask the child to continue coughing to try to expel the foreign object.	152	34.0
	Call emergency services and wait for help to arrive	23	5.1
	Encourage the child to breathe deeply to try to clear his airways	17	3.8
	Offer the child water or another drink to help him remove the foreign body	39	8.7
	Perform abdominal compressions (Heimlich maneuver) on the child to help clear the airway	216	48.3
Choking Incident, Nine-Year-Old, Unresponsive, Unconscious, Next Step You Perform	Call an ambulance and start CPR	195	43.6
	I ask another teacher to help me remove the foreign body	42	9.4
	Offer the child water or another drink to help him remove the foreign body	6	1.3
	Perform abdominal compressions for the baby to help clear the airway	143	32.0
	Transport the child to the nearest health center	61	13.6

Table 5 shows the association between knowledge levels about choking first aid and practical implications. Participants with high knowledge demonstrated a significantly higher rate of performing choking first aid (n=129) compared to those with poor knowledge (n=22) (p < 0.001). Additionally, a higher knowledge level corresponded to stronger support for mandatory first aid training for teachers (n=286) (p < 0.001). In a scenario involving a choking incident with a six-year-old, participants with greater knowledge were more likely to perform the Heimlich maneuver (n=249) (p = 0.039). However, for the scenario with an eight-year-old and a nine-year-old, no substantial difference was observed between the knowledge levels and practice. 

**Table 5:** Relationship between the knowledge level about choking and practice of choking first aid Frequency of choking first aid performed inside and outside of school showed a statistically significant finding (p-value<0.001) Importance of teachers attending first aid training for choking showed a statistically significant finding (p-value<0.001)

Practice on choking first aid		Knowledge about Choking First Aid		Sig. Value
		Poor Knowledge	High Knowledge	
Ever Performed Choking First Aid Inside/Outside of School	Don't Know	10	16	<0.001
	No	90	180	
	Yes	22	129	
Support to Make It Necessary for Teachers to Attend Course of First Aid for Choking	Strongly Disagree	1	2	<0.001
	Disagree	2	2	
	Not Sure	4	2	
	Agree	33	33	
	Strongly Agree	82	286	
Choking Incident of Six-Year-Old, Unable to Cry, Talk, Breathe, or Cough, Next Step You Perform	Ask the Child to Cough	23	44	0.039
	Call Emergency	11	15	
	Encourage the child to Breathe Deeply	3	12	
	Offer the child water to Remove the foreign body	6	5	
	Perform Heimlich Maneuver	79	249	
Choking Incident, Eight-Year-Old, Able to Speak, Cough Ineffective, Next Step You Perform	Ask the Child to Cough	39	113	0.290
	Call Emergency	10	13	
	Encourage the child to Breathe Deeply	5	12	
	Offer the child water to Remove the foreign body	14	25	
	Perform Heimlich Maneuver	54	162	
Choking Incident, Nine-Year-Old, Unresponsive, Unconscious, Next Step You Perform	Call Ambulance and Start CPR	46	149	0.294
	Took help from other teachers to remove the foreign body	15	27	
	Offer the child water/ drink to remove the foreign body	3	3	
	Perform Heimlich Maneuver	40	103	
	Transport the child to the nearest health center	18	43	

## Discussion

First aid knowledge is vital for immediate response to health emergencies, with choking being a life-threatening issue, especially in young children. Choking incidents often result from airway obstruction, posing significant risks if not promptly addressed. The findings of our cross-sectional study examining the knowledge, attitude, and practice of first aid management of choking among primary school teachers in Riyadh, Saudi Arabia provide significant insights into the preparedness and responses of educators in handling choking incidents.

Our study population consisted mainly of married, Saudi female teachers, with a substantial portion having over a decade of teaching experience. These demographic characteristics are consistent with the general profile of educators in the region, underlining the relevance of our findings to the local educational landscape.

There is a significant level of awareness among participants regarding the universal signs of choking, common behaviors leading to choking, and the signs of both complete and partial airway obstruction [2]. Notably, 73.8% of teachers claimed proficiency in providing first aid, with learning sources including social media (47.0%) and previous first aid training (18.1%). However, the confidence level was comparatively lower, with only 38.9% expressing confidence in their first aid abilities.

This finding highlights the importance of enhancing educators' knowledge and confidence in providing first aid [16]. While social media can be a valuable source of information, the variability in accuracy and reliability necessitates structured and evidence-based training programs [17]. These programs can address the specific needs of teachers in the school environment, offering them the confidence to act effectively during choking incidents.

The overwhelmingly positive attitudes of the participants toward the necessity of immediate action in choking emergencies emphasize their sense of responsibility and duty as educators. Previous studies show that a teacher is a first responder in case of any type of school-related emergency [18]. The inclination toward prioritizing knowledge of first aid, coupled with a strong belief in its life-saving potential, reflects the participants' acknowledgment of the urgency and significance of prompt intervention. However, the divided opinion on whether to call emergency services before initiating first aid signals a potential gap in understanding the critical timelines of choking management, necessitating focused educational interventions to address these misconceptions.

There is a mixed pattern of practices among the participants concerning first aid management of choking incidents. While a considerable percentage reported performing choking first aid, the proportion advocating for teachers to attend a first aid course indicates an awareness of the necessity for continuous training and skill development. Boada et al. (2022). showed that many countries have initiated campaigns to educate their citizens about the treatment and management of choking [19]. The inclination toward performing the Heimlich maneuver in the case of choking incidents demonstrates a basic understanding of first aid practice. Previous medical literature shows that this maneuver has over 80% success rate in emergency choking [20]. However, the varying responses in different scenarios suggest the need for standardized and consistent approaches to first aid management among educators.

The association between knowledge levels and practical implications further underlined the significance of knowledge in shaping the preparedness of educators to manage choking incidents. The significantly higher rate of performing first aid among participants with a higher knowledge level highlights the direct correlation between knowledge acquisition and practical application. Similarly, Ganfure et al. (2023) showed that previous first aid skills or training is associated with higher attitude and knowledge toward first aid [1]. 

Comparatively, our findings are consistent with existing literature on first aid preparedness among educators, emphasizing the critical role of adequate training and awareness in ensuring timely and effective responses to choking incidents. However, the specific focus on the Saudi Arabian context and the unique challenges faced by educators in the region distinguishes our study from previous research. By providing a comprehensive overview of the current knowledge, attitude, and practice among educators, our study contributes to the growing body of knowledge on first aid preparedness within the educational setting.

Despite the valuable insights gained from our study, several limitations include the cross-sectional design hindering causal inference and self-reported data introducing response biases. Geographical focus limits generalizability. Future research requires longitudinal designs and diverse participant samples for comprehensive insights into first aid preparedness among educators in varied contexts.

Recommendation 

The study recommended that there is a necessity to improve teachers' knowledge and also attitudes toward choking. This can be achieved through health education programs. The mass media and teaching directorate should provide a comprehensive program regarding choking at the beginning of the new academic year.

Follow-up studies should be conducted to assess the effectiveness and identify any gaps that need to be addressed in future awareness about choking first aid campaigns.

## Conclusions

Our study underscores the critical importance of tailored and comprehensive training programs aimed at enhancing the knowledge and skills of educators in managing choking incidents within school environments. By addressing the gaps identified in our study, policymakers and educational institutions can develop evidence-based strategies to improve the overall safety and well-being of students, thereby creating a more secure and conducive learning environment for all.
